# In Vivo Pre-Instructed HSCs Robustly Execute Asymmetric Cell Divisions In Vitro

**DOI:** 10.3390/ijms21218225

**Published:** 2020-11-03

**Authors:** Mukul Girotra, Vincent Trachsel, Aline Roch, Matthias P. Lutolf

**Affiliations:** 1 Institute of Bioengineering, Laboratory of Stem Cell Bioengineering, Ecole Polytechnique Fédérale de Lausanne (EPFL), CH-1015 Lausanne, Switzerland; vincent.trachsel@gmail.com (V.T.); alineroch.roch@gmail.com (A.R.); 2 Institute of Chemical Sciences and Engineering, School of Basic Sciences, EPFL, CH-1015 Lausanne, Switzerland

**Keywords:** hematopoietic stem cells, asymmetric cell division, paired daughter cells, single-cell analysis, in vivo activation, HSC fate choice, metabolism

## Abstract

Hematopoietic stem cells (HSCs) are responsible for life-long production of all mature blood cells. Under homeostasis, HSCs in their native bone marrow niches are believed to undergo asymmetric cell divisions (ACDs), with one daughter cell maintaining HSC identity and the other committing to differentiate into various mature blood cell types. Due to the lack of key niche signals, in vitro HSCs differentiate rapidly, making it challenging to capture and study ACD. To overcome this bottleneck, in this study, we used interferon alpha (IFNα) treatment to ”pre-instruct” HSC fate directly in their native niche, and then systematically studied the fate of dividing HSCs in vitro at the single cell level via time-lapse analysis, as well as multigene and protein expression analysis. Triggering HSCs’ exit from dormancy via IFNα was found to significantly increase the frequency of asynchronous divisions in paired daughter cells (PDCs). Using single-cell gene expression analyses, we identified 12 asymmetrically expressed genes in PDCs. Subsequent immunocytochemistry analysis showed that at least three of the candidates, i.e., Glut1, JAM3 and HK2, were asymmetrically distributed in PDCs. Functional validation of these observations by colony formation assays highlighted the implication of asymmetric distribution of these markers as hallmarks of HSCs, for example, to reliably discriminate committed and self-renewing daughter cells in dividing HSCs. Our data provided evidence for the importance of in vivo instructions in guiding HSC fate, especially ACD, and shed light on putative molecular players involved in this process. Understanding the mechanisms of cell fate decision making should enable the development of improved HSC expansion protocols for therapeutic applications.

## 1. Introduction

The life-long maintenance of a stable pool of hematopoietic stem cells (HSCs) and the concomitant production of committed daughter cells, giving rise to all mature blood cells, is achieved by a tightly orchestrated balance of HSC fate choices. This regulation restricts uncontrolled stem cell expansion or exhaustion that could be detrimental for an organism. Asymmetric cell division (ACD) is a powerful strategy that allows for, the maintenance of stem cell number, as well as the production of committed progeny, called hematopoietic progenitors, at the same time [1]. Although the majority of HSCs are present in a quiescent cell cycle state [2], the rapid turnover of blood production is ensured by highly proliferative progenitors which lack the capacity for long-term self-renewal but have full lineage differentiation potential. These multipotent progenitors (MPPs) produce mature blood cells via a multi-step differentiation process by first giving rise to lineage restricted progenitors that further divide to produce mature blood cells [3].

HSCs are located in niches composed of a complex array of different cell types and extracellular factors that play a key role in determining HSC fate [4,5]. The resident mesenchymal stem cells (MSCs) are the precursors of these different niche cells. Osteoblasts, derived from MSCs, in the endosteal niche have been shown to strongly support HSC function via both direct cell–cell interaction and secretion of growth factors [6,7,8,9,10,11]. Additionally, the perivascular niche containing MSCs, endothelial cells, and CXCL12-abundant reticular (CAR) cells has also been shown to influence HSC function [12,13,14,15]. Interestingly, bone marrow adipocytes, one of the downstream lineages of MSCs, have been previously shown to be negative regulators of HSC function [16] and have now recently been shown to support hematopoiesis by secreting stem cell factor (SCF) [17]. More recently, MSCs have also been derived from other areas of the body, such as the oral cavity [18] but their interaction with HSCs has still not been fully understood.

Classical studies on paired daughter cells (PDCs) arising from HSCs have revealed different colony forming potential [19,20] and in vivo reconstitution ability [21,22] in sister cells, suggesting the occurrence of ACD. More recently, Ap2a2, an endocytic gene, was shown to be involved in ACD of murine HSCs [23], and myosin II [24] and CD133 [25] emerged as regulators of ACD in human stem and progenitor cells (hHSPCs). Interestingly, the cellular metabolic machinery has also been implicated in ACD of HSCs. Long-term continuous imaging revealed asymmetric partitioning of lysosomes and autophagosomes upon HSC division, predicting an asymmetric fate of the two daughters [26], with low lysosomal activity corresponding to an improved reconstitution capability [27]. Additionally, asymmetric segregation of mitochondria was also believed to influence the fate of HSC daughters [28,29]. However, despite these intriguing findings, little is known about the mechanisms of ACD in HSCs. A major experimental challenge is that in the absence of key instructive signals of the in vivo niche, ACDs are rare events that consequently are exceedingly difficult to capture and study in vitro. Moreover, the frequency of ACD was found to be highly dependent on external factors such as cytokines used for in vitro cultures, making these fate choices a consequence of the culture condition used [20].

Here, we have tried to address these issues by ”pre-instructing” HSCs directly in their in vivo niche, in order to increase the frequency of ACD events, thus facilitating the systematic study of this important physiological process. Specifically, we used interferon alpha (IFNα) treatment to activate quiescent HSCs, i.e., to promote their exit from the dormant state in their native niche [30]. Intriguingly, single cell time-lapse microscopy showed that the extent and proportion of asynchronously dividing HSCs increased by nearly two-fold in IFNα-activated cells as compared with the unperturbed cells. Moreover, single-cell multigene expression analysis of PDCs revealed an asymmetrically expressed gene set in PDCs of IFNα-activated HSCs. We found HSC surface markers (such as CD34, CD150, and JAM3) and several metabolic intermediates (such as aconitase 1 (Aco1), glucose transporter 1 (Glut1), and hexokinase 2 (HK2)) to be highly asymmetrically partitioned in PDCs. Confocal imaging confirmed the asymmetric expression of JAM3, Glut1, and HK2 at the protein level. Finally, using co-staining and colony forming assays, we found that daughter cells with low Glut1 and high JAM3 expression were more primitive. Collectively, we established IFNα-based in vivo HSC activation as a useful approach to increase the frequency of ACD, enabling the study of the molecular mechanisms of this important process.

## 2. Results

### 2.1. In Vivo Activation of Hematopoietic Stem Cells (HSCs) by IFN Alpha Increases Asynchronous Cell Divisions

**Figure 1:**
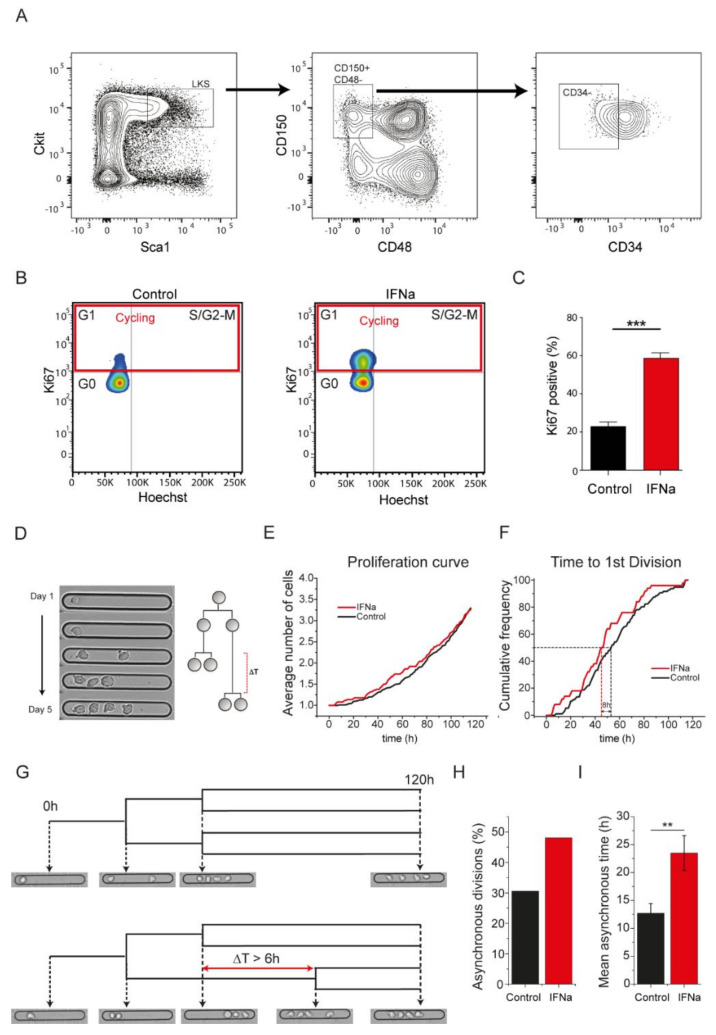
In vivo activation of hematopoietic stem cells (HSCs) by interferon alpha (IFNα) increases asynchronous cell divisions. (**A**) HSCs were freshly isolated from the bone marrow using Lin- cKit+ Sca1+ CD150+ CD48- CD34- markers; (**B**) Cell cycle analysis using Ki67 and Hoechst staining on freshly isolated HSCs from control and IFNα-treated mice. Cells in red box (Ki67 positive) constitute a fraction of HSCs in a cycling state (G1+S/G2-M phase); (**C**) HSCs isolated from IFNα-treated mice show a significant increase in the cycling fraction (Ki67 positive cells); (**D**) Single-cell proliferation kinetics analysis using live cell imaging of HSCs on a poly(2-hydroxyethyl methacrylate)-trimethylolpropane trimethacrylate copolymer (pHEMA-TMPTA) micro-groove platform; (**E**,**F**) Proliferation kinetics of live and dividing HSCs isolated from control and IFNα administered mice. The average proliferation over 5 days remains similar in the two conditions, with IFNα-activated cells carrying out the first division much faster (~8 h) as compared with control HSCs; (**G**) Representative example of synchronous division (top) and an asynchronous division (below) with ΔT > 6 h being used as a threshold for asynchrony; (**H**) IFNα-activated HSCs show a notable increase in the proportion of asynchronous divisions; (**I**) IFNα-activated HSCs show a significant increase of asynchronous time division between daughter cells. *** *p* < 0.001 and ** *p* < 0.01.

We used IFNα treatment of mice to promote the exit of HSCs from their dormant state [30,31]. Using the well-established combination of cell surface markers, Lin-ckit+ Sca1+ (LKS) CD150+ CD48-CD34- [2,12,32], we isolated long-term HSCs (hereafter referred to as “HSCs”) from IFNα-treated and control mice by flow cytometry-based cell sorting (Figure 1A). In line with published data [30,31], IFNα treatment resulted in a significant increase in the proportion of cycling (Ki67 positive) HSCs (Figure 1B,C), confirming that a large proportion of HSCs had exited their quiescent state upon IFNα exposure.

We next assessed the single-cell proliferation kinetics of freshly isolated HSCs, from control and IFNα-treated mice, using a hydrogel micro-groove array platform suited for single cell trapping and long-term tracking of rare cells such as HSCs [29]. Using this platform, we cultured cells in basal serum-free media and measured the single-cell division dynamics of 147 control and 87 IFNα-activated HSCs over a period of five days (Figure 1D–F). HSCs isolated from IFNα-treated mice carried out the first cell division markedly quicker as compared with the control HSCs (Figure 1F). After 53 h, half of the HSCs isolated from control mice had divided the first time as compared with 45 h in the case of IFNα-activated cells (Figure 1F), mirroring the differences in the proportion of dividing cells (labeled by Ki67) of both populations (Figure 1B,C). Importantly, the averaged proliferation kinetics (Figure 1E) over five days were similar among the two conditions, showing that acute IFNα treatment led to a transient activation and not an uncontrolled proliferation of HSCs, in line with previous literature [30]. Single-cell proliferation kinetics measured on a previously developed hydrogel microwell array platform (Figure S1A) [33,34,35] confirmed these results (Figure S1B,C).

We and others previously identified that a key attribute of HSCs was their slower cell cycling rate as compared with their closely related multipotent progenitors [2,34,36,37]. Therefore, asynchrony in division arising from differences in cell cycle length of PDCs has been previously used as a proxy for ACD [29]. Here, we assessed the difference in time of division of PDCs to define the synchrony of cell division (ΔT). A pair of daughter cells was defined as asynchronous if the ΔT was longer than 6 h [29] (Figure 1G). We observed that the proportion of asynchronous divisions was higher (Figure 1H) and the average asynchrony time was significantly longer in IFNα-activated HSCs (Figure 1I) as compared with the controls. A similar analysis of divisional asynchrony of HSCs grown on PEG microwells confirmed these results (Figure S1D,E). This increase in asynchrony suggested a possible increase in the frequency of asymmetric division where one daughter (presumably the slower cycling one) retained stem cell potential and the other daughter (presumably the faster cycling one) underwent commitment, supporting earlier observations for the production of two nonidentical daughter cells with different colony forming and blood reconstitution levels in vitro [19,20,21,22].

### 2.2. Identification of Asymmetrically Expressed Genes in Paired Daughter Cells (PDCs)

Previous studies have demonstrated that acute activation by IFNα promoted HSC self-renewal without the loss of stem cell potential [30,31]. Since IFNα-mediated activation occurred in the native bone marrow niche without loss of stem cell potential and promoted an increase in asynchronous divisions in vitro (Figure 1H), we hypothesized that it could serve as a suitable system to study in vivo niche-instructed HSC fate choices and specifically the process of ACD. To test this, we performed multigene expression analysis in PDCs and compared the gene expression profiles of sister cells. To this end, we isolated IFNα-activated HSCs from the bone marrow and cultured them as single cells in PEG microwells in a basal serum-free culture condition in order to follow their proliferation kinetics (Figure S2A). The PEG microwell system is an open system that allows easy access to cells for their collection [33,34]. Upon division, PDCs were isolated from individual microwells by micromanipulation (Figure S2B) and their gene expression profile was analyzed using single-cell quantitative RT-PCR. We shortlisted 47 candidate genes for expression analysis (described in experimental methods) on every cell. These genes belonged to various metabolic pathways or were earlier identified as important regulators of HSC function. All candidate genes were analyzed in 24 sets of PDCs. Strikingly, when Log_2_(Ct) values of the two cells were plotted against each other, several genes showed distinct patterns of expression. Qualitative analysis revealed that, on the one hand, some candidate genes demonstrated symmetric expression behavior, with almost all pairs found close to the diagonal (Figure S3A). On the other hand, various genes were asymmetrically expressed (Figure S3B), with one cell of the pair expressing the gene at higher levels (low Ct) and the other at lower levels (high Ct). However, for many of these potentially asymmetric genes, a subpopulation of pairs that appeared close to the diagonal (Figure S3B) remained, suggesting that not all pairs were carrying out ACD.

To identify the most asymmetrically expressed gene set, every gene was assigned a quantitative value to determine where it ranked as compared with the others. To this end, we obtained the average Δ*Ct* (difference in expression between two cells of a pair) for every gene across all pairs and designated it as the “gene score”.

(1) $$Gene\;score=\frac{1}{n}\left(\overset{n\;pairs}{\underset{i=1}{\sum }}(\Delta Ct)\right)\;where\;\Delta Ct=\left|Ct\;Cell_{a}-\;Ct\;Cell_{b}\right|$$

**Figure 2:**
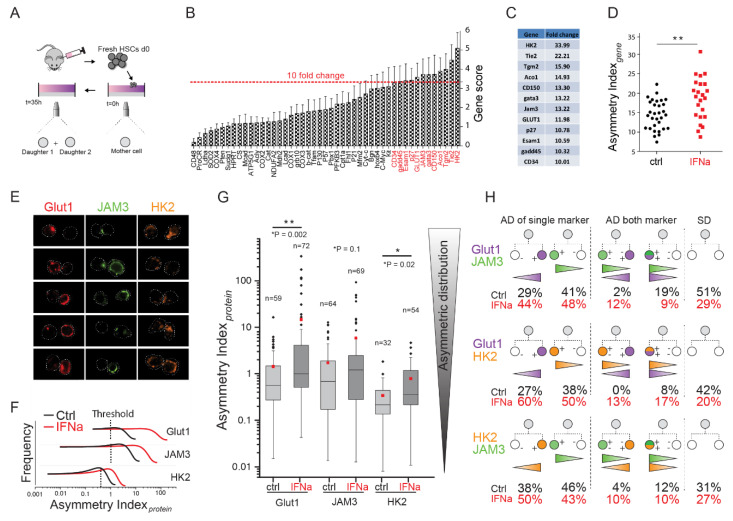
Identification of asymmetric gene and protein expression in paired daughter cells (PDCs). (**A**) Freshly isolated HSCs were tracked during culture until first division (35 h) using a time-lapse microscope. A microwell array platform was used for gene expression analysis and a micro-groove platform for protein expression analysis (Figure S2); (**B**) The gene score values show a wide variation from one gene to another. A stringent threshold (average 10-fold difference in gene expression between PDCs) was put to identify the “asymmetric gene set”; (**C**) Table showing 12 candidate genes that were seen to cross the 10-fold threshold (fold change values in table); (**D**) IFNα-treated PDCs show a higher asymmetry index as compared with control PDCs; (**E**) Representative immunofluorescent images showing asymmetric expression of Glut1, JAM3, and HK2 in 15 different PDCs; (**F**,**G**) Analysis of PDCs asymmetry for Glut1 (*n* = 59 control and *n* = 72 IFNα), JAM3 (*n* = 64 control and *n* = 69 IFNα treatment), and HK2 (*n* = 32 control and *n* = 54 IFNα) with IFNα-treated cells or control; (**F**) PDCs asymmetry in frequency with threshold defining “asymmetric protein expression” used in (**H**) Glut1 = 1, JAM3 = 1, HK2 = 0.3; (**G**) PDCs asymmetry. IFNα condition shows significantly higher levels of asymmetric index for Glut1 and HK2 as compared with the control. Boxplot’s interquartile range from the 25th to the 75th percentile. A line and red square show, respectively, median and mean, and the error bar is SD (outliers lower or upper inner fence is equal to 1.5 the interquartile range); (**H**) The protein expression pattern in PDCs (all PDCs considered) for Glut1, JAM3 and HK2. Asymmetry with one marker (first column) are shown in total percentage (without considering other markers). A double asymmetry (two asymmetric proteins) analysis is shown in the second column. An asymmetry comprising two proteins (for example “P1” and “P2”) on two daughter cells (defined by c_a_ and c_b_) is either “coinciding”, a cell having high expressions of both proteins “P1” “P2” (phonetically defined by $c_{a}{\;}_{P2}^{P1}{=}_{+}^{+},\;c_{b}{\;}_{P2}^{P1}{=}_{-}^{-}$), or “diametrical”, a cell having more than one protein “P1” but less than of “P2” $c_{a}{\;}_{P2}^{P1}{=}_{-}^{+}$ and, conversely, his sister more than one protein “P2” and less than “P1” $c_{b}{\;}_{P2}^{P1}{=}_{+}^{-}$. Number of pairs analyzed for co-staining as follows: *n* = 69 IFNα and *n* = 63 for control Glut1/JAM3, *n* = 30 IFNα and *n* = 26 for control Glut1/HK2, and *n* = 30 for IFNαa and *n* = 26 for control JAM3/HK2. ** *p* < 0.01, and * *p* < 0.05.

A gene score of 1 indicated an average two-fold difference in expression in the PDCs, which incremented to 4-, 8-, 16-, and 32-fold, with gene scores of 2, 3, 4, and 5, respectively. We found a huge range of this score with some genes as low as 0.195 (ca. 1.2-fold difference) and others as high as 5 (ca. 32-fold difference) (Figure 2B). We applied an arbitrary threshold of a 10-fold difference to identify a set of the most highly asymmetric genes from our candidate list; a set of 12 genes was found to be above this threshold (Figure 2B,C). This set included genes controlling different metabolic pathways such as glycolysis (Glut1, HK2) and TCA cycle (Aco1), as well as well-known HSC markers such as JAM3, Tie2, CD150, and CD34. Notably, the cell cycle kinase inhibitor p27 was also asymmetrically expressed, probably contributing to asynchrony in cell cycle of PDCs. The presence of metabolic candidates in the asymmetric gene set indicated the importance of cellular metabolism, especially glycolysis and mitochondrial pathways, in regulating HSC fate choices.

On the basis of this asymmetrically expressed gene set, we evaluated the asymmetry associated with each individual pair. To obtain a single quantitative readout of symmetry for a pair, we calculated the Euclidian distance between the two sister cells by summing up the square Δ*Ct* value for all genes in the “asymmetric” gene set and calculating its square root [34]. We termed this value the asymmetry index (A.I) for a pair, where a higher value of A.I reflected a greater asymmetry in expression.

(2) $$A.I_{gene}=\sqrt{\left(\overset{N\;genes}{\underset{i=1}{\sum }}{\left(Ct\;\left\{cell\;a\right\}-Ct\;\left\{cell\;b\right\}\right)}^{2}\right)}$$

Pairs with a high A.I value had asymmetric expression of these genes, and very often the expression was restricted to only one cell of the pair (Figure S3), while, in pairs with a low A.I value, most of the genes were symmetrically expressed and very close to the diagonal (Figure S3). A comparative analysis revealed that IFNα-activated pairs had significantly higher values of A.I than their control counterparts (Figure 2D), suggesting that it can be used as a suitable model to study the link between gene expression and asymmetric fate in HSCs.

### 2.3. Analysis of the Protein Expression of Glut1, JAM3, and HK2

After identifying a list of 12 highly asymmetrically expressed gene candidates, we analyzed whether this asymmetry applied to some of them at the protein level. We selected three candidates, namely Glut1, JAM3, and HK2, to check their expression in PDCs derived on the aforementioned micro-groove platform (Figure 1 and Figure S2C). Single HSCs were seeded onto the grooves and tracked by time-lapse microscopy. Upon division, PDCs were fixed and analyzed by immunocytochemistry (Figure S2C). Notably, the closed micro-groove platform allowed cell encapsulation without mixing the cells between different grooves during different staining steps, which was difficult to achieve with other systems [29]. Then, we compared the protein expression between sister cells (denoted by $c_{a}\;and\;c_{b}$) using confocal microscopy and obtained an index of asymmetry at the protein level, calculated as follows:

(3) $$A.I_{protein}=\frac{c_{a}-\;c_{b}}{c_{b}},\;\mathrm{with}\;c_{a}>c_{b}$$

Preliminary analysis revealed that some PDCs were very symmetric ($A.I_{protein}$ < 0.01) (Figure S4) while others were highly asymmetric (Figure 2E) ($A.I_{protein}$ > 100), both in the control and IFNα-treated cells. In total, we analyzed 131 pairs for Glut1, 133 for JAM3 and 86 for HK2. This analysis revealed that PDCs exhibited higher levels of asymmetry for Glut1, JAM3, and HK2 with IFNα treatment (Figure 2G), in line with the gene expression data (Figure 2B). In particular, Glut1 and HK2 showed significantly higher asymmetry, suggesting their importance in mediating ACD in HSCs. Although not significant, an increase in asymmetric JAM3 intensity was seen in IFNα−stimulated PDCs (Figure 2G). In line with our asynchrony (Figure 1H) and gene expression data (Figure 2D), we found an increase in the frequency of asymmetric distribution of all the three markers in the IFNα condition (Figure 2F).

To obtain a more comprehensive phenotype of PDCs, we co-stained individual cells for Glut1, JAM3, and HK2, applying a threshold of *A.I_protein_* equal to 1 (this corresponds to one sister having 100% more expression over the other) to identify asymmetric PDCs. However, since the overall asymmetry with HK2 was smaller, we lowered the threshold for HK2 to 0.3 (this corresponds to one sister having 30% more expression over the other) (Figure 2F). This analysis showed that, in the IFNα condition, Glut1 and JAM3 were more asymmetric individually (Figure 2H, top left panel). Next, we looked within these asymmetric pairs for double “diametrical” asymmetry and found it in 12% of IFNα PDCs (versus 2% in controls). This suggested that one daughter cell exhibited a higher expression of one marker and lower expression of the other as compared with its sister cell (i.e., Glut^high^JAM3^low^ and Glut^low^ JAM3^high^ PDCs) (Figure 2H, top middle panel). In contrast to diametrical asymmetry, only 9% of IFNα PDCs had a “coinciding” asymmetry (i.e., both markers being expressed more on the same daughter cell), and 19% in the control (Figure 2H, top middle panel). Interestingly, both markers were symmetrically expressed in more than half of the control PDCs (51%) and only 29% of IFNα PDCs (Figure 2H, top right panel). These data suggested a different signature in asymmetry expression in control and IFNα cells. Previous studies have shown higher JAM3 [34,38,39] and lower Glut1 expression [27,40] on functional HSCs. Therefore, we assumed that PDCs with a Glut1^low^ JAM3^high^ phenotype could represent a more primitive cell type as compared with other combinations.

Additionally, other combinations (i.e., Glut1/HK2 and JAM3/HK2) demonstrated an increase in single and double asymmetry in the IFNα-treated PDCs (Figure 2H).

### 2.4. Characterization of Glut1 and JAM3 as Asymmetric Cell Division (ACD) Markers

**Figure 3:**
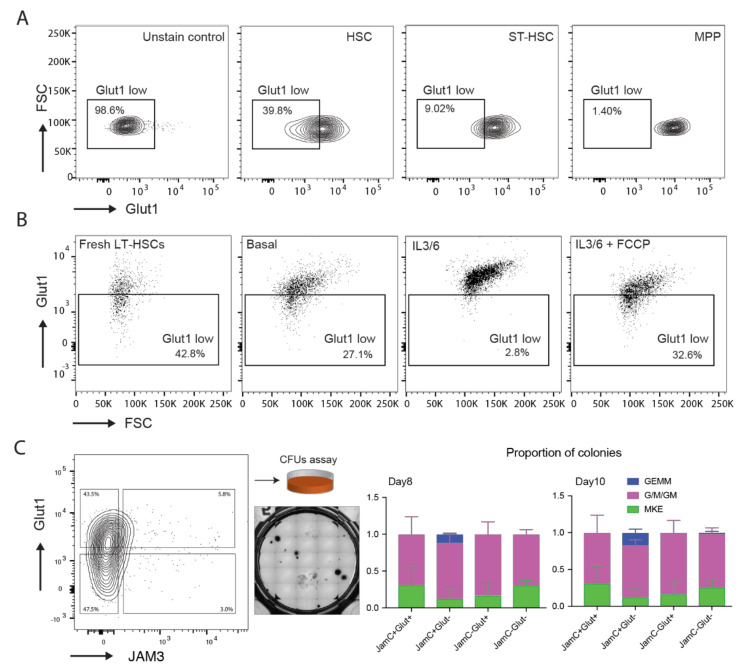
Characterization of Glut1 and JAM3 as markers for asymmetric division in HSCs. (**A**) Expression of Glut1 was tested in different populations of the hematopoietic hierarchy using flow cytometry. HSCs (HSCs, LKS CD150+ CD48-CD34-), short term HSCs (ST-HSCs, LKS CD150+ CD48-CD34+), and multipotent progenitors (MPPs, LKS CD150-) were identified using a combination of cell surface markers and stained with Glut1 to be analyzed by flow cytometry. Glut1 levels changed dramatically from HSCs (Glut1 low ~40%) to ST-HSCs (Glut1 low ~9%) to MPPs (Glut1 low ~1.5%), with concomitant increase as we move down in the hematopoietic hierarchy; (**B**) Glut1 expression in HSCs after 3 days of in vitro culture was assessed using flow cytometry. HSCs cultured in basal media show a small loss of Glut1 low fraction as compared with fresh uncultured HSCs. Differentiation inducing (IL3/6) condition shows a striking loss of Glut1 low fraction, with overall Glut1 levels going up. The addition of FCCP (mitochondrial uncoupler) to the IL3/6 condition reverts this loss of Glut1 low fraction and brings down the level of Glut1 expression; (**C**) HSCs were cultured for three days in basal media and were sorted for Glut1 and JAM3 post culture. The stem cell potential of these sorted cells was determined by their colony-forming unit (CFU assay) ability. Glut1 low JAM3 high cells show a significant increase in the proportion of more primitive (GEMM) colonies and concomitant decrease in the less primitive (Mk/E) colonies, suggesting Glut1 low JAM3 high cells are more primitive than other populations (i.e., Glut1 high JAM3 low, Glut1 high JAM3 high, Glut1 low JAM3 high). (CFU-)G, colony-forming unit-granulocyte; (CFU-)M, colony-forming unit-macrophage; (CFU-)GM, colony-forming unit-granulocyte, macrophage; (CFU-)GEMM, colony forming unit-granulocyte, erythrocyte, macrophage, megakaryocyte); Mk/BFU-E, megakaryocyte/burst-forming unit-erythroid.

Several previous studies have highlighted the importance of glycolytic metabolism in HSC fate determination [31,41,42,43]. Indeed, to determine if low Glut1 expression, as hypothesized above, was a hallmark of a more primitive hematopoietic population, first, we estimated Glut1 levels by flow cytometry in different cell populations of the hematopoietic hierarchy. Strikingly, we found a stepwise increase in the Glut1 expression from the most primitive population (HSCs, LKS CD150+ CD48-CD34-) to the more committed cell types (ST-HSC, LKS CD150+ CD48-CD34+ and MPP, LKS CD150-) (Figure 3A). Next, we assessed Glut1 expression in HSCs after in vitro culture and compared it with freshly isolated HSCs from the bone marrow. HSCs cultured in basal media showed a small decrease in the Glut1^low^ fraction (Figure 3B), while HSCs cultured in differentiation inducing conditions (containing IL3 and IL6 [31]) showed a large increase in Glut1 expression. This elevated Glut1 level suggested an increased glucose uptake to fuel glycolysis and mitochondrial oxidation as a hallmark of differentiation [31]. Intriguingly, the addition of the chemical uncoupler FCCP to these differentiation inducing conditions, previously shown to maintain stem cell potential [31], completely reverted the profile of Glut1 expression (Figure 3B).

To further characterize the ”diametrical” asymmetry that we observed in Glut1 and JAM3 (Figure 2H), we isolated the four populations by FACS, after in vitro culture, and plated them on methylcellulose to assess their colony forming ability. We found the Glut1^low^ JAM3^high^ cells to be the only cell type giving rise to the most primitive GEMM colonies (Figure 3C). These findings are in line with previous studies, showing high JAM3 and low Glut1 expression in functional HSCs [27,34,38,39,40].

## 3. Discussion

Here, we report a novel experimental strategy to analyze niche-instructed fate choices in HSCs. IFNα-mediated activation was used as a model to study ACD in murine HSCs. Live cell imaging revealed a significant increase in asynchronous divisions in activated HSCs, suggesting the execution of in vivo niche instructed ACDs. Previous studies in invertebrates have elegantly demonstrated the role of the niche in regulating ACD. For example, in *Drosophila*, a close association with the niche (that is, cap cell in the ovary and hub cell in the testis) was instrumental in the execution of ACD of germline stem cells. Instructive cues from the niche led to the activation of BMP (in the ovary) or JAK-STAT (in the testis) signaling, which eventually repressed differentiation in the daughter cell destined to maintain stemness [44,45,46,47]. However, carrying out such studies on single mammalian HSCs in the native bone-marrow niche was extremely challenging, therefore, researchers have resorted to PDC in vitro analysis. Although these conditions failed to recapitulate the bone-marrow niche, PDC analyses in HSCs have revealed different colony forming potential [19,20] and in vivo reconstitution ability [21,22] in sister cells, suggesting the occurrence of ACD. However, the precise mechanisms that regulate this fate choice are still not fully understood.

Our strategy to activate HSCs to initiate a division in the native niche, followed by single-cell gene expression analysis in PDCs, allowed the identification of an asymmetric gene set. Among them were genes that have been previously described to be important for HSC identification such as CD150 [12], Tie2 [6], and JAM3 [34]. Interestingly, Tie2 (the receptor for Ang1) has also been previously used as an asymmetry marker [48], validating our experimental approach for identifying asymmetry markers. Additionally, we found the cyclin dependent kinase inhibitor p27 to be asymmetrically expressed in PDCs. P27, in association with p57, has been demonstrated to be critical in regulating HSC cell cycle status [49]. Whether p27 asymmetry is the driver of more frequent asynchronous divisions in IFNα PDCs remains to be tested. We also identified metabolic genes such as Glut1, HK2, and Aco1 that were highly asymmetric in PDCs. Glut1 facilitates the transport of glucose across the cell membrane and was previously shown to be expressed at a higher level in multipotent progenitors as compared with HSCs [40]. Moreover, the glycolytic enzyme HK2, involved in the conversion of glucose to glucose-6-phosphate, was shown to be upregulated by HIF1a [50], a key component involved in HSC function [43]. The identification of these two candidates confirms earlier reports on the importance of glycolytic pathways in the regulation of HSC function [41]. The asymmetric expression of Aco1, an important TCA cycle enzyme in the mitochondria that converts citrate to iso-citrate, fits well with previous findings that implicate mitochondrial activity as a determinant of HSC fate [31,42].

For a long time, an increased glycolytic flux and a lower mitochondrial activity was believed to be a hallmark of HSCs. Interestingly, a recent study has challenged this dogma by demonstrating that functional HSCs have both a lower glycolytic flux, due to reduced Glut1 mediated glucose uptake, and reduced mitochondrial activity [27]. This overall lower metabolic activity profile allows these cells to reduce their cellular oxidative stress ensuring long-term functionality. Follow-up studies could resolve this issue by measuring the ratio of glycolytic to mitochondrial activity flux in HSCs.

Given the striking asymmetry observed in gene expression, we explored if this asymmetry was maintained at the level of protein expression. To this end, we employed a platform that allowed us to quantify protein expression in PDCs via immunofluorescence staining. This analysis revealed higher asymmetric protein partitioning of the three candidates in the IFNα condition. Flow cytometry analysis showed that Glut1 expression gradually increased as the cells became more specialized, in line with published data [40]. Furthermore, in vitro experiments showed that Glut1 expression was higher under differentiation culture conditions (IL3/6) as compared with stem cell maintenance conditions enforced by an uncoupler of mitochondrial oxidative phosphorylation (IL3/6 + FCCP) [31], suggesting a link between HSC fate and Glut1 expression.

At first, the lower expression of Glut1 appeared to be counterintuitive, as HSCs were believed to be more glycolytic as compared with progenitors [42,43,48]. However, HSCs are also known to express lower levels of global protein synthesis relative to progenitors [51], and were recently shown to maintain an overall lower metabolic activity profile [27]. We think that our data supports this notion.

Co-staining with two markers showed higher double asymmetry in IFNα-treated cells. Our in vitro functional analysis based on CFUs revealed that Glut^low^ JAM3^high^ was the most primitive subset in culture, in line with earlier reports showing lower Glut1 [40] and high JAM3 [34] in functional HSCs. Intriguingly, JAM3 was shown to be highly expressed on the surface of several niche cell types such as endothelial, perivascular, and mesenchymal cells located in the mouse bone marrow [34]. Moreover, HSCs cultured on artificial niches displaying JAM3 showed higher blood reconstitution capacity [34], confirming the importance of the niche in regulating HSC fate.

A delicate balance between self-renewal and differentiation is important in maintaining a constant pool of HSCs in the niche. Uncontrolled expansion of the HSC pool by symmetric self-renewal divisions brings with it an inherent risk of developing into cancer, while symmetric differentiation divisions can eventually lead to the exhaustion of the HSC pool, and therefore the existence of ACDs balances these two effects. Our data suggest that metabolism plays a key role in regulating this process in addition to conventional pathways. The identification of JAM3 and Tie2 as asymmetric markers indicates the importance of niche signals in the regulation of ACD. Whether HSCs are able to switch between symmetric and asymmetric divisions at the single cell level is still an open question and the mechanisms that control this switch are yet to be investigated. Interestingly, the aging hematopoietic system contains an expanded pool of HSCs, and it remains to be seen whether or not this expansion is due to amplified symmetric HSC divisions via intrinsic signals or to changes in the niche signals. Furthermore, it is not known whether these altered fate programs make aged HSCs more susceptible to cancer. Studies addressing these important questions may allow us to develop new approaches targeting specific pathways to achieve controlled HSC expansion for the clinic.

## 4. Materials and Methods

### 4.1. In Vivo Activation of HSCs

HSCs were activated to exit dormancy by interferon alpha (IFNα) treatment following published protocols [30,31]. Briefly, subcutaneous injections in C57Bl/6J mice were carried out with 10,000 U of IFNα (R&D systems, MN, USA) 48 and 24 h prior to bone marrow extraction. Control mice were injected with an equivalent volume of the vehicle (PBS + 0.1% BSA).

### 4.2. Antibodies

The following antibodies were used: cKit-PeCy7 (2B8, Biolegend, CA, USA), Sca1-APC (D7, Biolegend, CA, USA), CD150-PeCy5 (TC-15-12F12.2, Biolegend, CA, USA), CD48-PB (GM48-1, Biolegend, CA, USA), CD34-FITC (RAM34, eBiosciences, CA, USA), and SAV-PO (Life Technologies, CA, USA). A mixture of biotinylated mAbs against CD3, CD11b, CD45R/B220, Ly-6G, Ly-6C, and TER-119 was used as lineage depletion cocktail (BD Biosciences, NJ, USA).

### 4.3. Flow Cytometry and Cell Sorting

Flow cytometry analysis of hematopoietic stem and progenitor cells was performed on freshly isolated bone marrow (BM) from 8–12-week-old C57Bl/6J mice. BM was extracted from crushed femora, tibia, and hip bone. The, the cell suspension was filtered through a 70 μm cell strainer and erythroid cells were eliminated by incubation with red blood cells lysis buffer (eBioscences, CA, USA). Lineage-positive cells were removed with a magnetic lineage depletion kit (Miltenyi Biotech, Germany). Cell suspensions were stained with a panel of specific antibodies for stem and progenitor cells and FACS-sorted on BD FACS Aria II. The hematopoietic stem cell (HSC) compartment was identified and sorted with the following cell surface phenotype: Lin- Ckit+ Sca1+ (LKS) CD150+ CD48- CD34-.

### 4.4. Cell Cycle Analysis

FACS sorted HSCs were fixed and permeabilized using a Cytofix/Cytoperm plus kit (BD Biosciences, NJ, USA), according to the manufacturer instruction. The, cells were stained overnight with Ki67 FITC (BD Biosciences, NJ, USA) at 4 °C, and 10 min with Hoechst 33,342 (Invitrogen, CA, USA). Stained cells were run on a BD LSRII for cell cycle analysis.

### 4.5. Micro-Groove Array Platform Fabrication

Micro-groove arrays were fabricated and used, as previously described [29]. Briefly, the platform was composed of two parts, i.e., one containing the groove array (bottom) and one used as a lid to close the groove (top). The lid covalently reacted with the bottom part, therefore, cells seeded on the groove array were encapsulated allowing an unambiguous tracking of cells and their progeny. The bottom part was made of poly(2-hydroxyethyl methacrylate)-trimethylolpropane trimethacrylate copolymer (pHEMA-TMPTMA) and the permeable lid of self-assembling polyethylene glycol (PEG).

### 4.6. PEG Microwell Array Fabrication

PEG microwells were formed at the bottom of 96-well microplate (BD) or 4-well plate (Nunc) by crosslinking 4arm-PEG-thiol (PEG-SH, 10 kDa) with 8arm-PEG-vinboneulfones (PEGVS, 10kDa) at 5% *w*/*v*. Hydrogel films were micropatterned by soft embossing with PDMS (polydimethylsiloxane, VWR) stamps for one hour to create an array of microwells, as described previously [33,34,35].

### 4.7. Single-Cell Proliferation Analysis by Time-Lapse Microscopy

Freshly isolated HSCs were seeded on the platforms. Platforms were directly placed on the time-lapse microscope (Zeiss Axio Observer Z1 inverted microscope equipped with a motorized stage) for image acquisition. An incubation chamber maintaining temperature and CO_2_ levels allowed for live cell imaging. The stage was programmed to scan the microwell array surface and acquire images of multiple positions every 3 h (30 min or 1 h for the micro-groove platform) for the duration of the culture period. Single-cell proliferation kinetics were assessed based on time-lapse movies.

### 4.8. HSC Cultures

All HSC cultures were maintained at 5% CO_2_ at 37 °C in basal media, Stemline II (Sigma, MO, USA) supplemented with 100 ng/mL SCF (R&D Systems, MN, USA), 2 ng/mL Flt3 ligand (R&D Systems, MN, USA), and 0.5% P/S. Differentiation media was made by supplementing basal media with 20 ng/mL IL3 (R&D Systems, MN, USA) and 100 ng/mL IL6 (R&D Systems, MN, USA). For uncoupler experiments, 5 μM carbonyl cyanide 4-(trifluoromethoxy) phenylhydrazone (FCCP) (Sigma, MO, USA) was added to the differentiation medium every 24 h.

### 4.9. Single-Cell Proliferation Analysis

Individual cells cultured in microwells were imaged on a Zeiss Axio Observer Z1 inverted microscope equipped with a motorized stage. An incubation chamber maintaining temperature and CO_2_ levels allowed for live cell imaging. The stage was programmed to scan the microwell array surface and acquire images of multiple positions every 3 h. Single-cell proliferation kinetics were assessed based on time-lapse movies.

### 4.10. Micromanipulation of Paired Daughter Cells for Single-Cell Analysis

Single cells in microwells having undergone one division to give rise to two daughter cells were identified after 40 h in culture. Paired daughter cells were isolated by micromanipulation in 20 μm diameter micro capillaries (Eppendorf). Single cells were ejected from the micro capillaries into lysis solution for subsequent single-cell PCR.

### 4.11. Selection of Candidate Genes

In total, 47 candidate genes, listed in Table 1, were selected for expression analysis in PDCs. Some of those genes were identified in a microarray analysis where they were differentially expressed in HSCs and compared with MPPs [52], or compared with mobilized or leukemic HSCs [53]. These included, key cell surface molecules such as JAM3, Tie2, ProCR, and Esam1; intracellular adaptor proteins Grb10 and Fhl1; and, cycling dependent kinase inhibitor p57 [54]. After a careful review of published work, we included cell cycle genes p21, p27, and p130, which were shown to be important in HSC quiescence [49,55,56]; HSC maintenance genes b-cat [57], Pten [58], and Gata3 [59]; and, important self-renewal mediators, HoxB4 [60] and c-myc [61,62]. With recent developments in the field that are strongly implying the role of metabolism in HSC fate choices, we included a variety of metabolic genes for our analysis. These included, a glucose transporter (Glut1), key glycolytic enzymes (HK2, PFKFB3, Ldha) [31,43], enzymes involved in TCA cycle (CS, Acyl, Aco1, Suclg1, Mdh2), and some key components of the oxidative phosphorylation machinery. According to recent work that implicated fatty acid oxidation as a key to HSC maintenance [48], we included two acyl-coA dehydrogenases (MCad and Lcad) and an enzyme that facilitates the entry of fatty acids into the mitochondria (Cpt1a). With a growing amount of information on the detrimental effects of ROS on HSC function, we included two key antioxidants (SOD2 and Cat) that were shown to be instrumental in keeping ROS levels low in HSCs [43,63].

### 4.12. Single-Cell qRT-PCR

Micromanipulated single cells were ejected in 0.2 ml PCR tubes containing 10 μL of lysis solution (9 μL single cell lysis solution + 1 μL single cell Dnase I). Cells were incubated in the lysis solution at RT for up to 30 min, followed by addition of 1 μL of single-cell stop solution and incubation at RT for up to 20 min. Then, the samples were stored at −20 °C. Reverse transcription and pre-amplification were performed sequentially on the lysed cell sample using a Single Cell-to-Ct Kit (Life Technologies, CA, USA). Conditions for reverse transcription were 10 min at 25 °C, 60 min at 42 °C, and 5 min at 42 °C. Gene Expression TaqMan Assays (Life Technologies, CA, USA) corresponding to the gene set (Table 1) were pooled and diluted at 0.2X in 1X TE buffer pH 8.0 for pre-amplification. Samples were incubated for 10 min at 95 °C and pre-amplified for 14 cycles of 15 s at 95 °C and 4 min at 60 °C on a thermal cycler. The pre-amplified samples were diluted 1:15 in 1X TE buffer pH 8.0 and stored at −20 °C. Real-time quantitative PCR was performed with Gene Expression TaqMan Assays (Life Technologies, CA, USA) on a 7900 HT system (Applied BioSystems, CA, USA). Conditions for amplification were 2 min at 50 °C and 10 min at 94.5 °C followed by 40 cycles of 5 s at 97 °C and 1 min at 59.7 °C. Expression values over the threshold of the machine were set to 40 (ct = 40).

### 4.13. Immunostaining and Images Analysis in the Multigroove Platform

At the end of the culture period, cells were fixed (4% PFA) and permeabilized (0.1% TritonX) before blocking with 10% goat serum on the platform. Immunostaining was performed using Glut1-AlexaFluor 647 (1:500) (clone EPR3915, cat no ab195020, Abcam, United Kingdom), JAM3-FITC (1:100) (clone CRAM-18 F26, cat no MCA5935F BioRad, CA, USA), and HK2 (1:500) (clone 4H1, cat no ab76959, Abcam, United Kingdom) with a secondary antibody AlexaFluor 555 (1:500) (cat no A-21425 Thermofisher Scientific, MA, USA). Z-stacks of paired-daughter cells were obtained using a confocal spinning-disk microscope (NikonTi, Crest spinning disk with ANDOR EMCCD camera). Sum of pixels value of z-stack images were calculated to obtain the total protein expression of a cell. Image analysis was performed with ImageJ.

### 4.14. Glut1 Staining for FACS Analysis

Freshly sorted/cultured cells were stained with anti Glut1-PE (polyclonal cat no NB110-39113PE, Novus Biologicals, CO, USA) for 1 h, at 4 °C, followed by washing with PBS. Stained cells were run on a BD LSRII for analysis.

### 4.15. CFU Assay

Freshly isolated LT-HSCs were cultured for 3 days and directly stained with Glut1-PE (polyclonal cat no NB110-39113PE, Novus Biologicals, CO, USA) and JAM3-FITC (clone CRAM-18 F26 cat no MCA5935, BioRad, CA, USA) antibody. Stained cells were resorted based on Glut1 and JAM3 low and high signals and 35 collected cells of either population were plated in complete M3434 methylcellulose (Stem Cell Technologies, BC, Canada) following the manufacturer’s instructions. Colony-forming cell mass was scored on day 8 and 10 using STEMvisionTM (an automated colony counting machine, Stem Cell Technolgies, BC, Canada). Colony categories (erythroid and/or megakaryocyte progenitor cells CFU-MK/E, granulocytes and/or macrophage progenitor CFU-M/G/GM, and multipotential progenitors (CFU-GEMM) were evaluated by visual inspection.

### 4.16. Statistics

Data were statistically analyzed using Student’s t-test, Mann–Whitney test, and the Spearman’s rank correlation coefficient was determined for correlation analysis.

## Figures and Tables

**Table 1:** List of genes tested in single-cell multigene expression analysis.

Classification	Gene Name	Classification	Gene Name
Housekeeping gene	HPRT	Glycolysis	HK2
ECM proteins	Tgm2	Glycolysis	PFKFB3
ECM proteins	Bgn	Glycolysis	Glut1
Membrane proteins	Esam1	Glycolysis	Ldha
Membrane proteins	Tie2	TCA cycle	CS
Membrane proteins	JAM3	TCA cycle	Acly
HSC markers	CD150	TCA cycle	Aco1
HSC markers	CD48	TCA cycle	Suclg1
HSC markers	CD34	TCA cycle	Mdh2
HSC markers	C-kit	Oxidative phosphorylation	Cyt-C
Intracellular adaptors	Grb10	Oxidative phosphorylation	NDUFA2
Intracellular adaptors	ProCR	Oxidative phosphorylation	COX2 (Sdhd)
Intracellular adaptors	Fhl1	Oxidative phosphorylation	ATP5g1
Intracellular adaptors	b-catenin	Oxidative phosphorylation	COX1
Cell cycle regulators	P57 (cdkn1c)	Oxidative phosphorylation	COX3
Cell cycle regulators	P27 (cdkn1b)	Oxidative phosphorylation	COX4
Cell cycle regulators	P21 (cdkn1a)	Mitochondrial biogenesis	Mfn2
Cell cycle regulators	P130 (rab3gap)	Mitochondrial biogenesis	Tfam
Cell cycle regulators	Pten	Antioxidants	SOD2
Transcription factors	Pbx1	Antioxidants	Catalase
Transcription factors	Gata3	Fatty acid oxidation	MCad
Transcription factors	c-myc	Fatty acid oxidation	LCad
Transcription factors	Hoxb4	Fatty acid oxidation	CPT1a
DNA repair	Gadd45	-	-

## Supplementary Materials

The following supplementary figures are available online at https://www.mdpi.com/1422-0067/21/21/8225/s1.
