# Supplementary material for: In Vivo Pre-Instructed HSCs Robustly Execute Asymmetric Cell Divisions In Vitro
Source: Int J Mol Sci. 2020 Nov 3;21(21):8225. doi: 10.3390/ijms21218225 (PMC7663432; doi:10.3390/ijms21218225)
Supplement: Supplementary file 1 [file ijms-21-08225-s001.pdf]

Supplementary Figures:

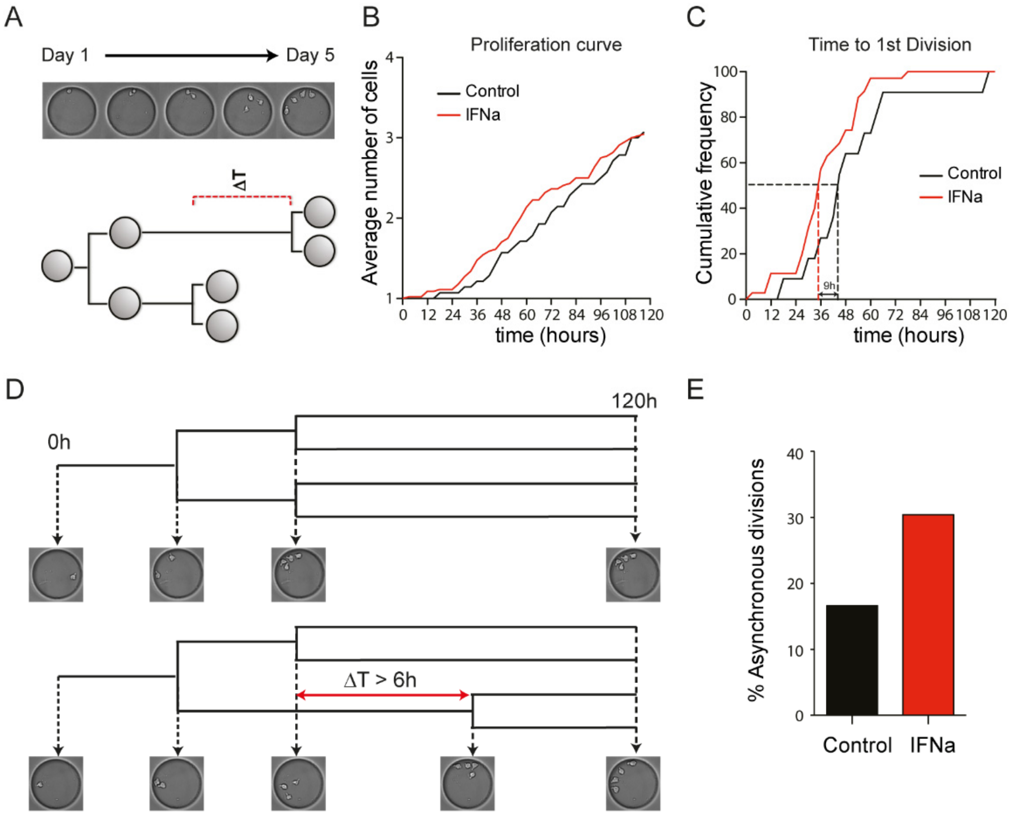

Figure S1. In vivo activation of HSCs by IFNa increases asynchronous cell divisions. (**A**) Single cell proliferation kinetics analysis using live cell imaging of HSCs in PEG microwells. (**B-C**) Proliferation kinetics of HSCs isolated from control and IFNa administered mice. The average proliferation over 5 days remains similar in the two conditions, with IFNa activated cells carrying out the first division much faster (~9h) as compared to control HSCs. (**D**) Representative example of synchronous division (top) and an asynchronous division (below) with ΔT 6 hours being used as a threshold. (**E**) IFNa activated HSCs show an increase in the proportion of asynchronous divisions.

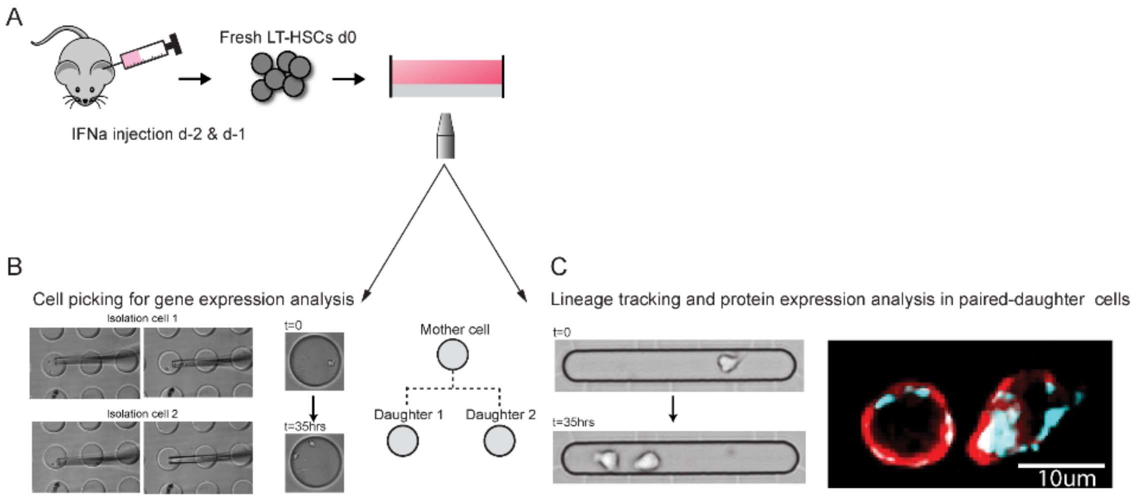

Figure S2. Gene and protein analysis of PDCs from IFNa-activated HSCs. (A) Freshly isolated HSC were cultured onto PEG microwells (B) or pHEMA-TMPTMA micro-grooves (C) and allowed to carry out a division. (B) Micromanipulation of paired daughter cells (PDCs) generated from a single HSC after one division, for multi-gene expression analysis using PEG microwell platform. (C) Protein expression analysis of PDCs generated from a single HSC after one division using pHEMA-TMPTMA micro-groove platform.

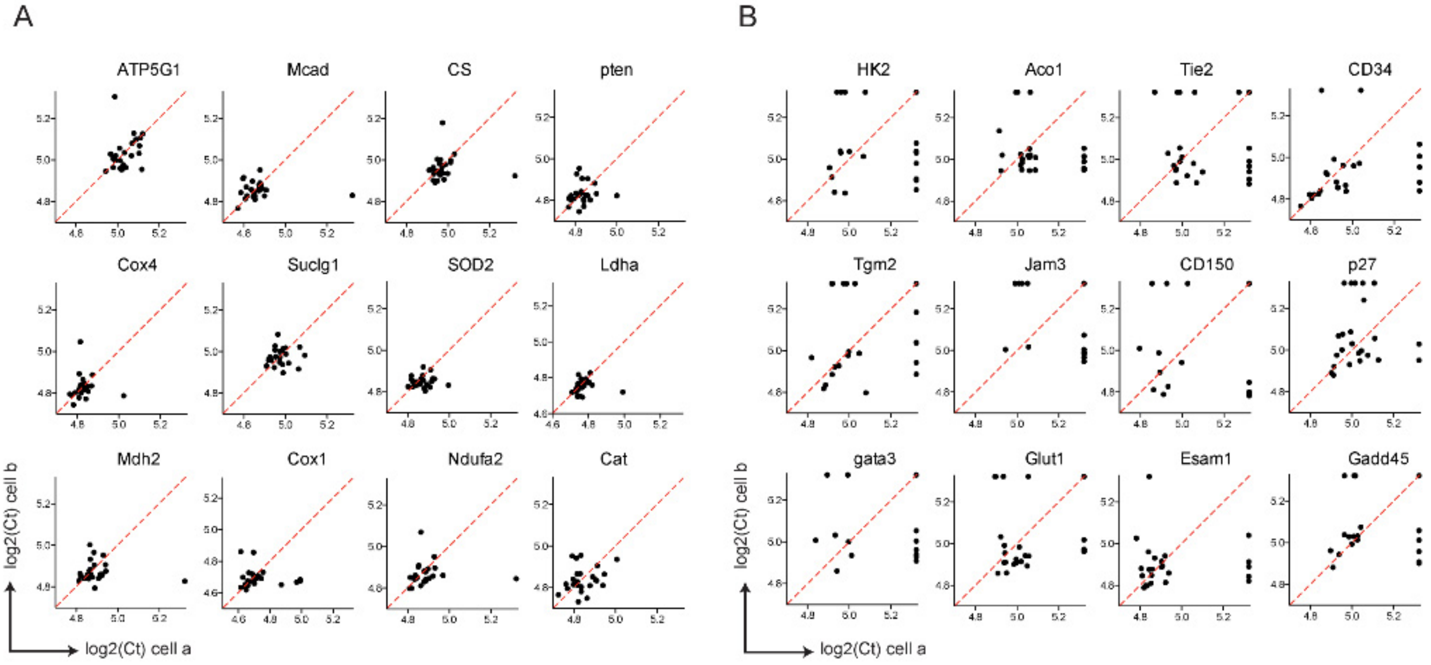

Figure S3. Qualitative analysis of distribution of gene expression in PDCs. Each gene is shown for the expression of 24 pairs of cells, with each dot representing a pair. (**A**) Representative examples of genes showing symmetric expression across most pairs. Most pairs appear close to the "symmetric diagonal" (in red). (**B**) Representative examples of genes showing asymmetric expression in pair daughter cells. Most pairs appearing far away from the "symmetric diagonal" (in red). Interestingly most of these genes have a subpopulation of pairs that show symmetric behaviour.

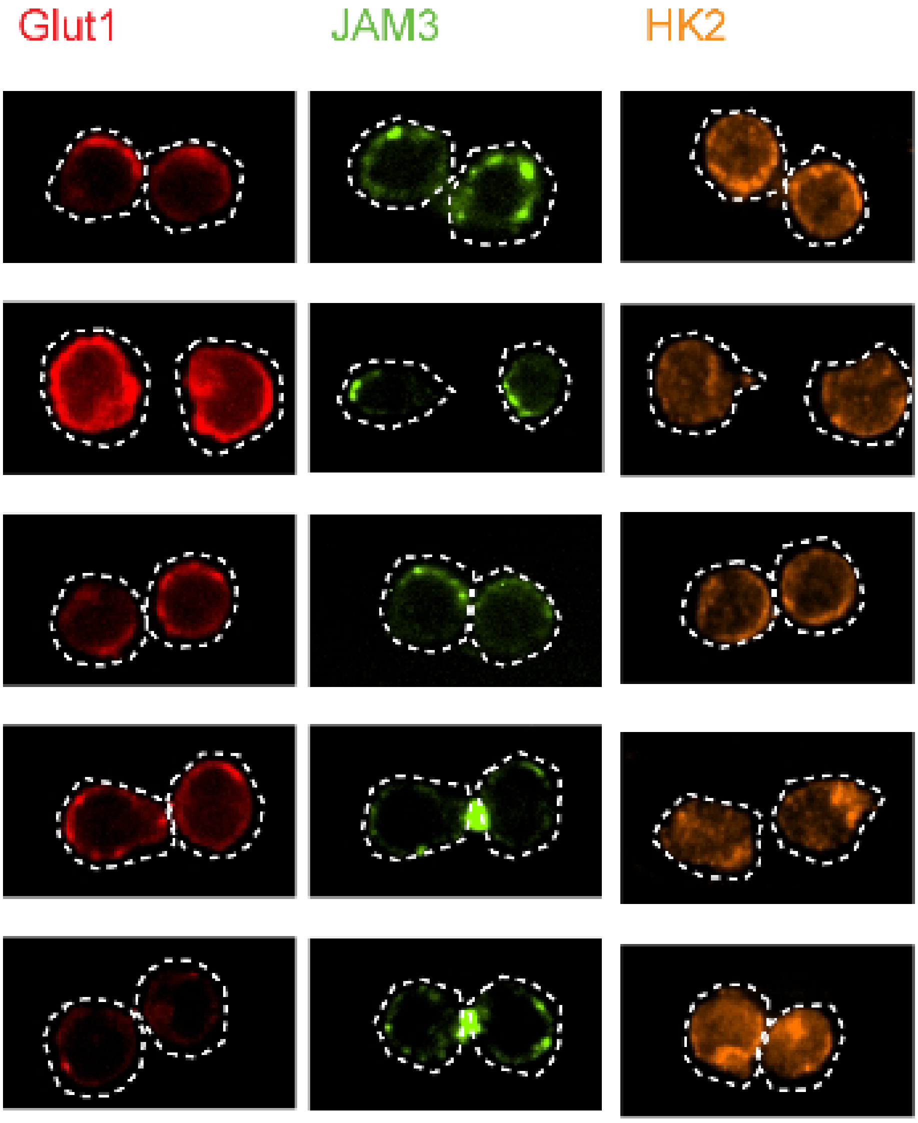

Figure S4. Representative examples of symmetric expression of Glut1, JAM3 and HK2 in paired daughter cell pairs.
